# A distinct sortase SrtB anchors and processes a streptococcal adhesin AbpA with a novel structural property

**DOI:** 10.1038/srep30966

**Published:** 2016-08-05

**Authors:** Xiaobo Liang, Bing Liu, Fan Zhu, Frank A. Scannapieco, Elaine M. Haase, Steve Matthews, Hui Wu

**Affiliations:** 1 Departments of Pediatric Dentistry and Microbiology, University of Alabama at Birmingham School of Dentistry, Birmingham, AL, USA; 2 Department of Life Sciences, Imperial College London, London, UK; 3 Department of Oral Biology, University at Buffalo, Buffalo, NY, USA

## Abstract

Surface display of proteins by sortases in Gram-positive bacteria is crucial for bacterial fitness and virulence. We found a unique gene locus encoding an amylase-binding adhesin AbpA and a sortase B in oral streptococci. AbpA possesses a new distinct C-terminal cell wall sorting signal. We demonstrated that this C-terminal motif is required for anchoring AbpA to cell wall. *In vitro* and *in vivo* studies revealed that SrtB has dual functions, anchoring AbpA to the cell wall and processing AbpA into a ladder profile. Solution structure of AbpA determined by NMR reveals a novel structure comprising a small globular α/β domain and an extended coiled-coil heliacal domain. Structural and biochemical studies identified key residues that are crucial for amylase binding. Taken together, our studies document a unique sortase/adhesion substrate system in streptococci adapted to the oral environment rich in salivary amylase.

Gram-positive cell wall anchored proteins play important roles in bacteria-host interactions and disease pathogenesis. This family of proteins mediates diverse cellular functions through binding to various host receptors^1^,^2^,^3^. Many surface proteins are covalently linked to the cell wall by sortases^4^, membrane-associated cysteine transpeptidase enzymes that catalyze cleavage of a conserved cell wall sorting signal (CWSS), such as the LPXTG motif, within the C-terminus of their cognate substrates, and subsequently attach the cleaved substrates to peptidoglycan^5^. Sortase deficiency attenuates bacterial virulence in diverse infection models^6^,^7^,^8^,^9^.

Sortase homologs and their substrates have been classified into four groups based on sequence homology and substrate recognition in Gram-positive bacteria^4^,^10^. Sortase A (SrtA) is often referred to as a housekeeping sortase. It anchors the majority of LPXTG containing proteins to the bacterial cell wall. Accessory sortases, including sortase B (SrtB)^11^,^12^, sortase C^13^,^14^ and sortase D^15^,^16^ either anchor their specific substrates to the bacterial cell wall or assemble cell surface pili^16^,^17^,^18^,^19^,^20^. Many genes coding for accessory sortases, found in the same gene operons that encode their substrate proteins^12^,^14^,^16^,^21^,^22^, recognize a variety of C-terminal CWSSs within different substrates, which is crucial for the cell surface display.

Oral streptococci are primary colonizers of dental biofilms in the oral cavity^23^. Like other Gram-positive bacteria, oral streptococci possess numerous cell wall anchored proteins that contain typical housekeeping CWSS mediated by SrtA. Oral streptococci have also evolved to bind the most abundant salivary protein, amylase, which contributes to the development of the dental biofilms^24^. Amylase binding streptococci only colonize hosts that produce amylase in their saliva^25^, indicating a specific bacterium-host interaction. In *Streptococcus gordonii,* amylase binding is mediated by amylase-binding protein A (AbpA)^26^. Mutation of AbpA causes deficient biofilm formation and bacterial adhesion *in vitro*^27^. However, AbpA lacks the classic CWSS and is released into the culture supernatant^26^, but is also found on the cell surface of *S. gordonii*^28^. Moreover, binding of amylase by AbpA in *S. gordonii* differentially regulates expression of genes involved in fatty acid biosynthesis, and alters bacterial survival in stress conditions^29^. The precise mechanisms underlying these complex and diverse phenomena remain unknown.

*Streptococcus parasanguinis* and other amylase-binding streptococci (including *S. gordonii)* encode a unique two-gene cluster consisting of *abpA* and an accessory sortase, *srtB*. In this report, we demonstrate that AbpA is processed and anchored to the bacterial cell wall by SrtB. We have also solved the high-resolution solution structure of AbpA by NMR, determined key residues important for amylase binding, and identified the sub-domain that contributes to appearance of the AbpA ladder profile. This sortase-AbpA system is only found in selected species of oral streptococci, and is distinct from previously reported sortase and substrate systems. Thus, the current study should shed new insights on the bacterium-host interaction, which could suggest novel approaches for modulating the oral microbiota.

## Results

### A genetic locus consisting of *abpA* and *srtB* is highly conserved in oral streptococci

*S. parasanguinis* is a primary colonizer of the oral cavity. It has the ability to bind to salivary amylase. A gene coding for a putative amylase-binding protein (AbpA) is located upstream of a gene encoding a putative sortase B (SrtB). This *abpA-srtB* gene organization is highly conserved in oral streptococcal genomes (Fig. S1A), but not found in any other bacterial species.

**Figure 1:**
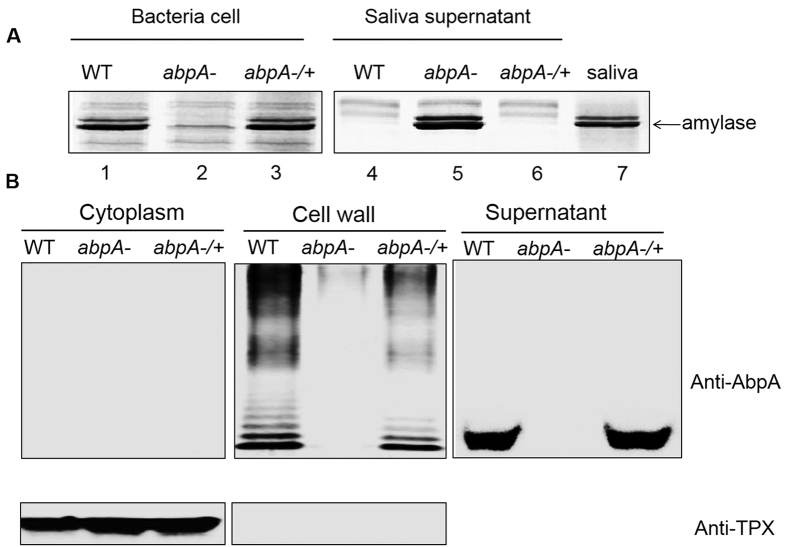
AbpA binds to amylase and is anchored to the bacteria cell wall. (**A**) *abpA* mutation abolished amylase binding. Bacterial cells harvested from *S. parasanguinis* wild-type strain, *abpA* mutant and the complemented strain (*abpA*−/+) were incubated with saliva for amylase binding assay. The intact bacterial cells (left) were separated from saliva supernatants (right), and subjected to SDS-PAGE analysis and protein staining. An arrow labels amylase. (**B**) AbpA is anchored to the cell wall. Subcellular fractionation was prepared from *S. parasanguinis* wild-type strain, *abpA* mutant and the complemented strain (*abpA*−/+) and examined by Western blot analysis using anti-AbpA antibody. TPX, a cytoplasmic protein, was used as a control.

Salivary amylase is known to bind to amylase-binding streptococci, with amylase completely removed from saliva following incubation with the bacterial cells^30^. Thus, the ability of a strain to bind to salivary -amylase was determined by examining the level of amylase in saliva supernatant after incubating bacterial cells with human saliva. Inactivation of *abpA* in *S. parasanguinis* rendered the bacteria unable to bind amylase, causing the enzyme to remain in the saliva supernatant (Fig. 1A, lane 2). Subsequent complementation restored wild-type levels of binding (Fig. 1A lanes 1 and 3, 4 and 6). Comparison of AbpA homologs revealed that significant sequence homology exists in both N-terminal signal sequence and C-terminal region (Fig. S1B). The C-terminal region contains the LPKTS motif followed by a short tail (AVK). It is distinct from reported classical cell wall sorting signals, which are much longer and contain the LPXTG motif, a hydrophobic domain and a charged tail (Fig. S1C).

### AbpA is anchored to the cell wall and presents as a ladder profile

To verify whether AbpA is anchored to the cell surface, we determined the subcellular localization of AbpA. AbpA was detected in both culture supernatant and cell wall fractions, but not in the cytoplasm (Fig. 1B). Tpx, a known cytoplasmic protein, was used as a positive control^31^. Detection of Tpx in the cytoplasm, but not in the cell wall, verified the purity of these subcellular fractions (Fig. 1B). Interestingly, a protein ladder profile was evident for AbpA only in the cell wall fraction but not in the supernatant, suggesting that AbpA is displayed on the cell wall as a higher order structure.

### SrtB mediates the cell surface localization of AbpA and amylase binding

**Figure 2:**
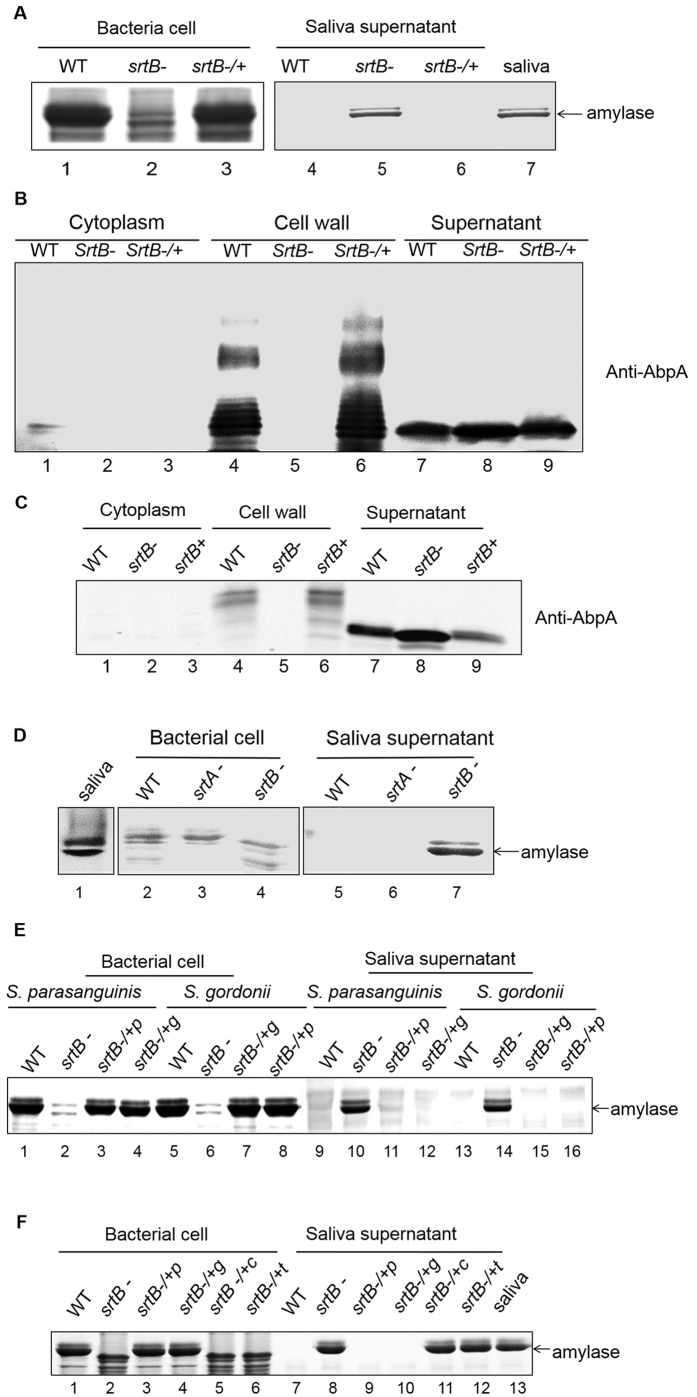
SrtB mediates the cell surface localization of AbpA and amylase binding. Amylase binding to bacterial cells was performed as described in Fig. 1. Amylase bound to the bacterial cells (left) and remained in the saliva supernatants (right) were analyzed by SDS-PAGE and Coomassie Blue protein staining. (**A**) *srtB* mutation abolished amylase binding. (**B**) *S. parasanguinis* and (**C**) *S. gordonii srtB* mutation abolished the cell wall display of AbpA. Subcellular fractions of wild-type strain, *srtB* mutant and the complemented strain were subjected to Western blot analysis with corresponding anti-AbpA antibody. (**D**) *srtA* mutation did not affect amylase binding. (**E**) Cross-complementation between *S. parasanguinis* SrtB and *S. gordonii* SrtB. Amylase was indicated by an arrow. +p, from *S. parasanguinis*; +g, from *S. gordonii*. (**F**) SrtB from *S. aureus* (*srtB* −/+*c*) and *S. pneumoniae* (*srtB* −/+*t*) cannot restore amylase binding of *srtB* mutant from *S. parasanguinis*. +p, from *S. parasanguinis*; +g, from *S. gordonii*; +c, from *S. aureus*; +t, from *S. pneumoniae*.

Given the fact that *abpA* and *srtB* genes are adjacent on the chromosome and AbpA was found on the cell wall, we hypothesized that SrtB mediates the anchorage of AbpA to the cell surface. Indeed, a SrtB-deficient mutant mimicked the AbpA mutant in terms of failing to bind amylase to the cell (Fig. 2A, lane 2 versus lanes 1 and 3). In addition, AbpA was released into the supernatant, and absent from the cell wall fraction when *srtB* was inactivated (Fig. 2B, lanes 5 and 8). The same protein distribution pattern was also seen in *S. gordonii* (Fig. 2C, lanes 5 and 8). Moreover, inactivation of *srtA*, a gene coding for the housekeeping sortase did not affect amylase binding (Fig. 2D, lanes 3 and 6), suggesting SrtB, not SrtA, enables the amylase binding through the presentation of AbpA on the cell surface.

We next tested whether SrtB from different oral streptococci would cross-complement each other. Indeed, the *srtB* mutant could be complemented by *srtB* from either *S. parasanguinis* or *S. gordonii* (Fig. 2E, lanes 4 and 8, 12 and 16), suggesting that SrtB from these species is functionally conserved. In contrast, SrtB from *S. aureus* or *S. pneumoniae* failed to complement the *srtB* mutant of either *S. parasanguinis* or *S. gordonii* (Fig. 2F, lanes 5 and 6, 11 and 12), suggesting that SrtB from these oral streptococci represents a distinct subfamily of B-class sortases. Sequence comparison of these sortases also supported this notion (Fig. S1D).

### SrtB polymerizes AbpA *in vitro*

**Figure 3:**
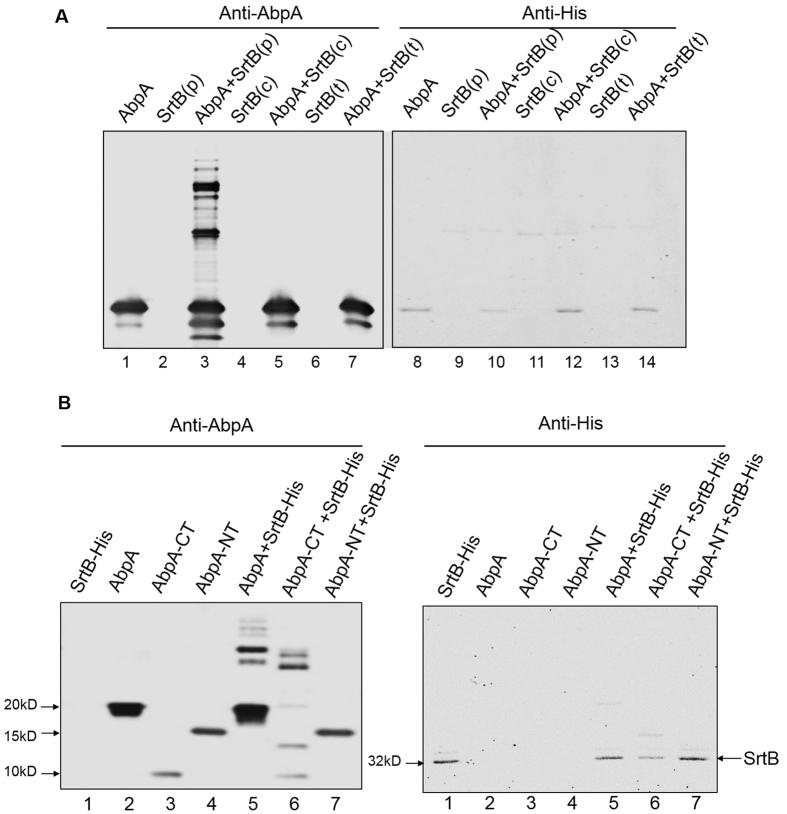
SrtB processes AbpA *in vitro*. (**A**) SrtB of *S. parasanguinis* processes AbpA *in vitro.* Recombinant AbpA-His was incubated with SrtB from different species and then probed with anti-AbpA and anti-His by Western blot analysis. (**B**) C-terminus of AbpA is crucial for the formation of a higher order of the AbpA structure. Recombinant AbpA and its variants were incubated with SrtB-His and then probed with anti-AbpA and anti-His by Western blot analysis. 1, SrtB-His; 2, AbpA; 3, AbpA-CT (139-207aa); 4, AbpA-NT (24-138aa); 5, AbpA + SrtB-His; 6, AbpA-CT (139-207aa) + SrtB-His; 7, AbpA-NT (24-138aa) + SrtB-His.

Since our *in vivo* studies suggest that SrtB anchors and processes AbpA (Fig. 2B, lanes 4 and 6), we established an enzymatic assay to determine whether SrtB can process AbpA *in vitro*. A simple protein ladder profile was visualized only when recombinant AbpA of *S. parasanguinis* was co-incubated with SrtB (Fig. 3A, lane 3), suggesting that oligomerization of recombinant AbpA is SrtB-dependent, albeit in a pattern different from that observed *in vivo*. To assess the specificity of SrtB, we overexpressed recombinant SrtB from *S. aureus* and *S. pneumoniae* that failed to complement *srtB* mutants of oral streptococci, and then used these reagents in the *in vitro* assay. No ladder profile for *S. parasanguinis* recombinant AbpA was detected, suggesting these SrtB sortases are unable to process AbpA *in vitro* (Fig. 3A, lanes 5 and 7). These data are consistent with our *in vivo* studies, further validating the specificity of SrtB. To determine which region of AbpA is important for producing the ladder profile, AbpA was split into two fragments, the N-terminal (24-138aa) and C-terminal (139-207aa) regions, and subjected to *in vitro* enzymatic studies with SrtB. The protein ladder profile was observed only when the C-terminal domain or the full-length AbpA was used (Fig. 3B, left panel, lanes 6 and 5), indicating that the C-terminal region harbors the motif(s) responsible for generating the ladder profile.

**Figure 4:**
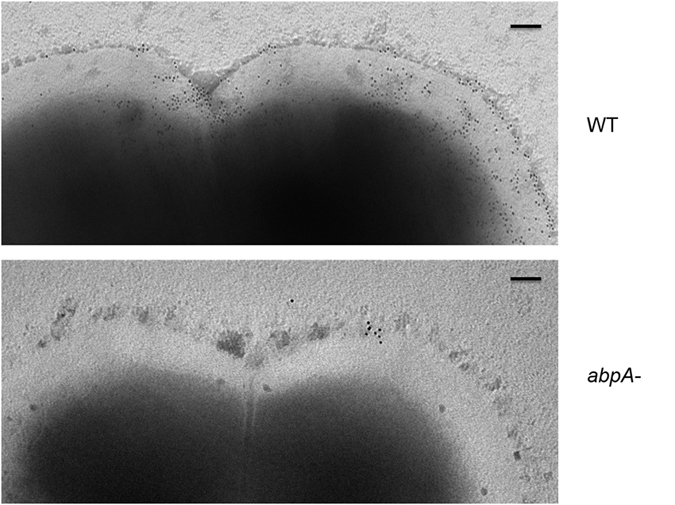
Localization of AbpA on *S. parasanguinis* cell. Cells were immobilized on honey-carbon grids and stained with antibody against AbpA, followed by goat anti-rabbit IgG conjugated to 6-nm gold particles. Grids were stained with 1% phosphotungstic acid and viewed with an electron microscope. (Scale bars: 0.1 μm).

To further examine whether the ladder profile of AbpA would translate into a higher order of AbpA polymer on the bacterial cell surface, immunogold labeling electron microscopy (EM) was used to visualize AbpA (Fig. 4). It is apparent that AbpA is presented and likely covalently localized on the cell surface, and mostly accumulates in the septal area, which resembles the localization of AbpA from *S. gordonii*^28^. These data do not distinguish whether AbpA is presented as monomers, polymers or both on the cell surface. It is conceivable that pilus-like polymers of AbpA could be present at a significantly lower abundance than monomers or that these species are flexible and collapse onto the bacterial surface during EM sample preparation.

### The C-terminal conserved domain is essential for AbpA anchoring and amylase binding

**Figure 5:**
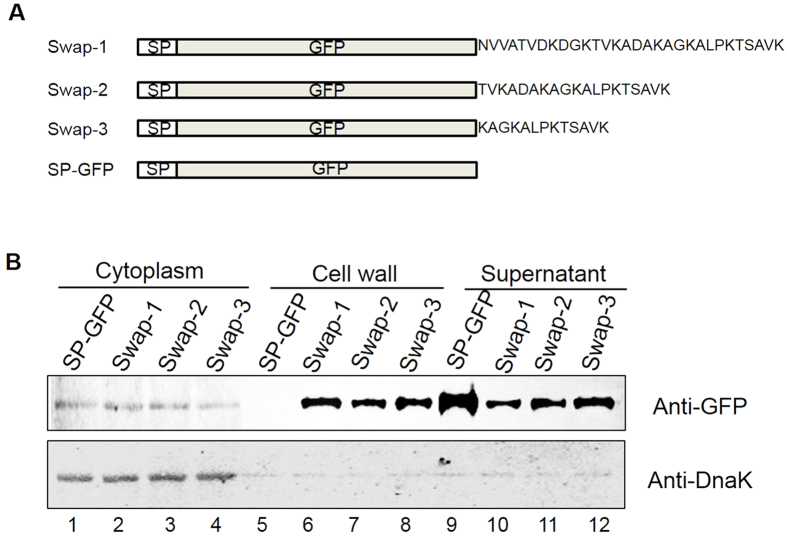
The C-terminal conserved domain is essential for anchoring of AbpA to the cell wall. (**A**) Diagram of the Swap constructs. GFP was fused after the signal sequence, and N-terminal to varying lengths of C-terminal region. Construct with no C-terminal domain was used as a control. (**B**) Subcellular localization of recombinant GFP proteins. Subcellular fractions of recombinant GFP from different *S. parasanguinis* strains were prepared and examined by Western blot analysis. DnaK detected by anti-DnaK antibody was used as a loading control.

To determine the requirement of the C-terminal conserved motif of AbpA for cell wall anchoring, we carried out domain swapping experiments by constructing a variety of recombinant AbpA constructs that contain an AbpA signal sequence, the green fluorescent protein (GFP) and a variety of C-terminal tail lengths, and then evaluated the subcellular distribution of recombinant GFP fusion proteins (Fig. 5A). The strain in which GFP was fused to the signal peptide (SP-GFP) without a C-terminal tail was used as a negative control. Without the conserved C-terminal domain, GFP was found released into the culture supernatant (Fig. 5B, lane 9) and could not be detected in the cell wall fraction (Fig. 5B, lane 5). However, in all three AbpA-GFP swap strains with varying sizes of the C-terminal tail, GFP was readily detected in both cell wall (Fig. 5B, lanes 6–8) and supernatant fractions (Fig. 5B, lanes 10–12). These data confirm that the C-terminal KAGKALPKTSAVK motif acts as the cell wall sorting signal to anchor the protein to the cell wall.

### Key residues in C-terminus are important for AbpA anchoring to the cell wall

**Figure 6:**
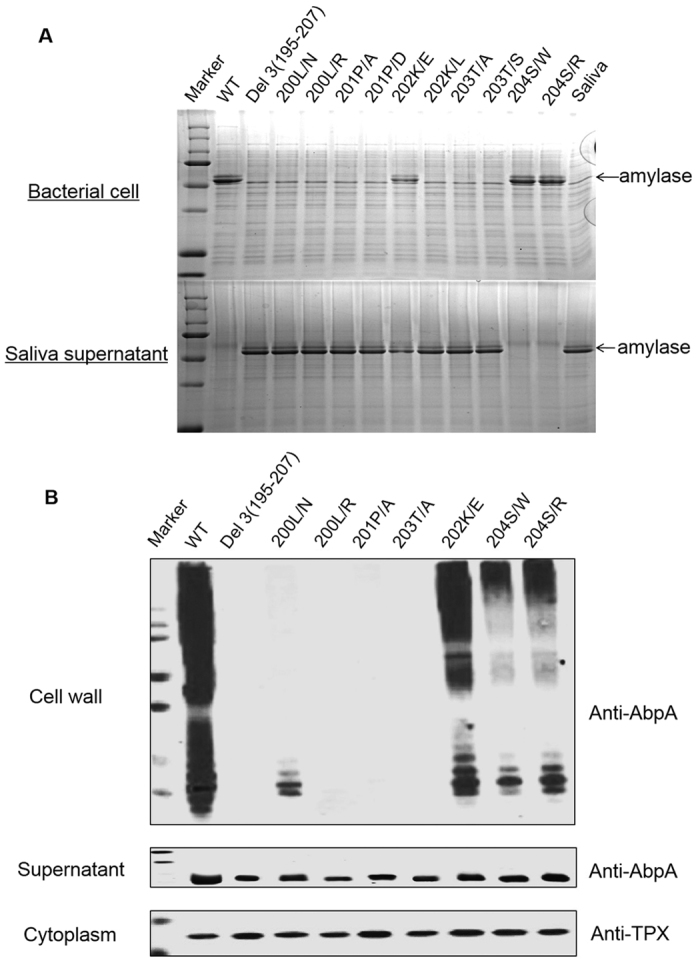
The C-terminal conserved domain is essential for amylase binding and anchoring to the cell wall. (**A**) Site-directed mutagenesis of the LPKTS motif was applied to precisely assess the role of key residues. Cell pellet of *S. parasanguinis* wild-type strain and the complemented *abpA* variants were incubated with saliva for amylase binding. Samples from the cell lysate and supernatant were analyzed by SDS-PAGE. (**B**) Subcellular localization of *S. parasanguinis* wild-type strain and the complemented *abpA* variants were examined by Western blot analysis.

To further determine the conservation of the C-terminal motif in anchoring AbpA to the cell wall, a series of deletion and site directed mutant strains were constructed and evaluated (Table S3 and Fig. 6). Three deletion mutants, devoid of either the entire conserved KAGKALPKTSAVK domain, the LPKTSAVK domain or only 3 amino acids AVK all lost amylase binding (Table S3). We then targeted the LPKTS motif more precisely with site-directed mutagenesis to assess the role of key residues. Amino acid residues LP within this motif are essential, as substitution of either L or P abolished amylase binding and the ladder profile on the cell wall (Fig. 6). Although substitution of K with L abolished amylase binding, the K to E substitution retained amylase binding (Fig. 6A). Substitution of T with either A or S completely abolished amylase binding and the ladder profile of AbpA on the cell wall, while substitution of S had minimal effect (Fig. 6). In addition, the mutations (200L and 201P) that abolished amylase binding and presumably cell wall anchoring also blocked the ladder profile (Fig. 6), suggesting the anchoring is prerequisite for the polymerization. These results also indicate the L and P residues within the LPKTS motif are conserved and crucial; however, K can be substituted by a similar residue and S is the most variable residue.

### SrtB specifically cleaves AbpA between T and S

**Figure 7:**
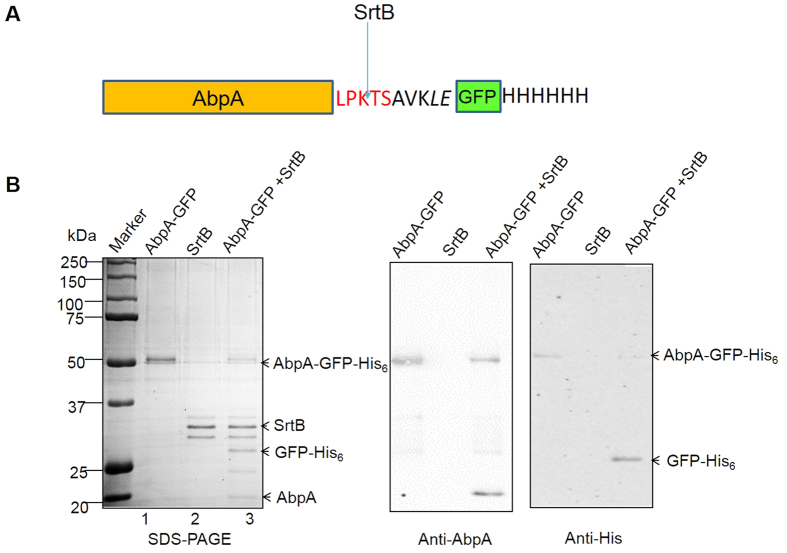
SrtB cleaves AbpA between T and S. (**A**) Diagram of a construct for the fusion protein AbpA-GFP-His_6_. (**B**) SDS-PAGE and Western blot analysis of enzymatic reactions with AbpA and SrtB *in vitro*. The reactions and appropriate controls were probed with anti-His and anti-AbpA antibody.

Canonical sortases cleave between T and G within the LPXTG motif, and subsequently anchor the cleaved peptide ending with T to peptidoglycans. To identify the SrtB cleavage site on AbpA, we generated a recombinant AbpA in which a GFP construct followed by a 6xHis tag was fused to the C-terminal LPKTSAVKLE (Fig. 7A). This construct allowed us to identify the cleavage site by performing N-terminal protein sequencing of the nickel affinity purified product after the cleavage by SrtB. When AbpA-GFP-6xHis co-incubated with SrtB, additional protein bands other than input proteins, SrtB and AbpA were evident (Fig. 7B, Lane 3 versus lanes 1 and 2), suggesting that an active cleavage occurred. After nickel affinity chromatography, a 28-kDa polypeptide was purified from the reaction and N-terminal sequencing revealed the first four amino acids were SAVK (data not shown), demonstrating that the cleavage site in the LPKTS motif lies between T and S.

### AbpA comprises two domains arranged in a novel elongated structure

**Figure 8:**
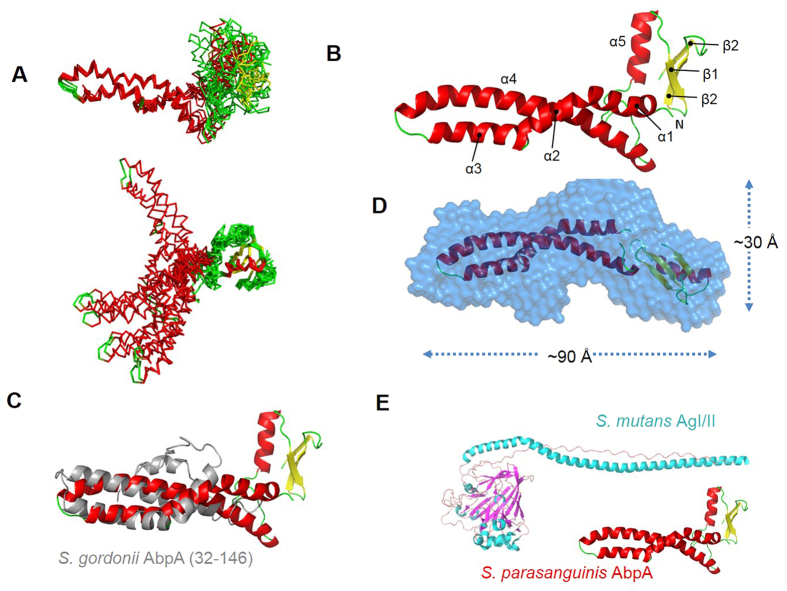
NMR and SAXS structure of AbpA (PDB ID: 2ND4). (**A**) Superimposition of the ten lowest energy NMR structures of *S. parasanguinis* AbpA over the N-terminal coiled-coil domain (top) and the C-terminal α/β domain (bottom). (**B**) Cartoon representation of the NMR structure of *S. parasanguinis* AbpA. The β-strands (β1–3) are colored in yellow and the five helices (α1–5) in red. Positions of the secondary structure elements are indicated on Fig. S1B. (**C**) Superposition of the N-terminal coiled-coil domains from the solution structure of *S. parasanguinis* AbpA and *S. gordonii* AbpA (PDB ID: 2MXX, residues 32-146). RMSD is 3.1 Å over 79 Cα atoms. (**D**) SAXS derived electron density for *S. parasanguinis* AbpA with the solution structures of N- and C-terminal domains separated and rigid body fit into the envelope and refined with RDCs. (**E)** Comparison of AntigenI/II of *S. mutans* and AbpA of *S. parasanguinis*.

To provide a more detailed insight into the structure, architecture and amylase binding of *S. parasanguinis* AbpA, we solved the solution structure of AbpA by NMR (Table 1 and Fig. 8A,B). Initial analysis of NMR line-widths for full-length AbpA reveals that the C-terminal 10 amino acid residues that encompass the LPKTS motif are flexible in solution, therefore final data collection and structural calculation were performed on an AbpA construct lacking this region (referred to AbpA herein). The structure reveals two distinct domains: an extended N-terminal helical coiled-coil region (25–131) and a small globular C-terminal domain (144–197) comprising a mixed α/β fold. Recently, the structure of truncated AbpA of *S. gordonii* was reported^32^, which revealed four principal helices between resides 45–145 that are folded into a similar anti-parallel coiled-coil arrangement as *S. parasanguinis* AbpA (Fig. 8C). In *S. parasanguinis* AbpA helical coiled-coil is extended relative to that observed for *S. gordonii* AbpA and the C-terminal region adopts a small globular fold, which in *S. gordonii* AbpA is ill-defined and largely unfolded^32^. Furthermore, the structure of *S*. *gordonii* AbpA failed to identify amylase-binding motifs. Even two major regions of AbpA (24-56aa and 124-165aa) were suggested to be required for *S. gordonii* binding to amylase^33^, the key motifs are still unclear. Therefore, the mode of amylase-binding by *S. gordonii* AbpA remains unknown.

To explore the inter-domain orientation of *S. parasanguinis* AbpA, we used small angle X-ray scattering (SAXS). After gel filtration chromatography of recombinant AbpA, SAXS measurements were recorded (Table 2). The SAXS density indicated that AbpA exists as an elongated, multi-domain structure with dimensions of approximately 90 × 30 × 30 Å, and the N- and C-terminal domains from our solution structure AbpA fitted well into the two halves of the SAXS density (Fig. 8D) and confirmed the overall extended shape. Although a DALI^34^ search failed to identify any similar structures deposited in the protein data bank, *S. parasanguinis* AbpA resemble the overall fibrillary topology of the A_3_VP_1_ fragment of *S. mutans* AgI/II (Fig. 8E)^35^. This structure comprises a central globular domain, which is flanked by an N-terminal alanine-rich region that adopts an extended α-helix and a C-terminal polyproline type II (PPII) helix. These two distinct helical features interact to form a 155 Å long stalk. It has been proposed that elongated helical stalk projects the apical tip from the bacterial surface for interaction with its host receptor salivary agglutinin (SAG) and a subsequent additional interaction occur within the C-terminal region close to the LPXTG sorting motif, which is responsible for anchorage to the cell wall^35^. The apical globular domain is not present in AbpA while the mixed α/β domain is located adjacent to the LPKTS motif. This arrangement would suggest that receptor binding likely involves the helical coiled coil of AbpA.

### Key residues crucial for AbpA binding to amylase

To explore the amylase-binding interface on AbpA, we initially performed an NMR titration experiment with human α-amylase to locate the interface on our refined solution structure. ^1^H-^15^N HSQC spectra were recorded on a ^15^N-labelled AbpA sample before and after step-wise addition of human α-amylase. Spectra were subsequently analyzed for perturbations in peak positions and line-widths, which would indicate the amylase-interaction surface on AbpA. A global decrease in the intensity of all AbpA resonances was observed upon the addition of amylase (Fig. S2A). At a 1:1 stoichiometric ratio the spectrum was undetectable indicating that a large multimeric complex had formed. This is consistent with the observation that the addition of amylase to culture supernatants of *S. gordonii* results in a precipitate^36^.

Although these global effects on the NMR spectrum of AbpA were the most striking, additional subtle, specific line-broadening effects could be observed in the early stages of the titration, which could indicate residues proximal to the amylase-binding surface. Most of the residues that broadened in presence of low concentrations of amylase, localized to a central region within N-terminal coiled-coiled domain.

**Figure 9:**
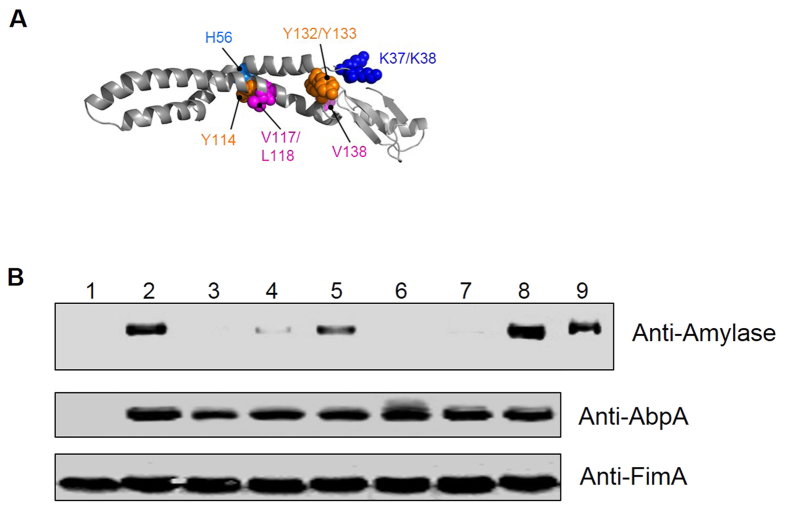
Key residues crucial for amylase binding. (**A**) Residues on the helical coiled coil region of AbpA were chosen for site-directed mutagenesis. (**B**) Mutation of key residues K37/K38A, Y132/Y133A, V117/L118A abolished amylase binding. Cell pellets of *S. parasanguinis* wild-type strain, *abpA* mutant and the complemented AbpA variants were incubated with saliva for the amylase-binding assay. Samples from cell lysates and supernatants were examined using anti-Amylase and anti-AbpA antibodies by Western blot analysis. FimA was used as a loading control. 1, *abpA*−; 2, *abpA*−/+; 3, K37K/38A; 4, H56A; 5, Y114A; 6, V117/L118A; 7, Y132/Y133A; 8, V138A; 9, Saliva.

Based on proximity to the NMR mapped surface as well as surface accessibility, we chose a selection of residues on the helical coiled coil region (Figs 8B and 9A) for site-directed mutagenesis and subsequent functional tests. Mutant variants were transformed into the *abpA* mutant of *S. parasanguinis* and subsequently evaluated for amylase binding. We also produced the AbpA mutant protein variants and examined their structural integrity and *in vitro* amylase binding by NMR. All mutant variants produced comparable level of engineered AbpA proteins in the native host (Fig. 9B, middle panel). However, K37/K38A, H56A, V117/L118 and Y132/Y133A variants failed to enable amylase binding while Y114A reduced the binding, demonstrating the role of these residues in the amylase binding (Fig. 9B upper panel). NMR spectra for these mutants were consistent with full-folded proteins and titrations with amylase confirmed the absence of an interaction with human α-amylase (Fig. S2B).

### NMR evidence for the interaction of AbpA with SrtB

**Figure 10:**
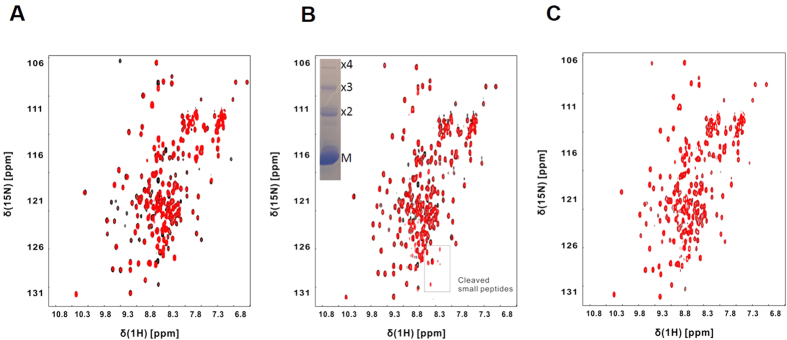
NMR titration of SrtB to AbpA. (**A**) Overlay of ^1^H-^15^N HSQC spectrum of AbpA alone (LPKTS motif - black) with that after the addition of one equivalent of SrtB (Red). (**B**) Overlay of ^1^H-^15^N HSQC spectrum of AbpA alone (LPKTS motif - black) with that after the addition of equivalent amount of SrtB and incubated for 2 h (red). Box indicates the appearance of peptide cleavage products likely from the C-terminal LPKTS motif. An inset of the SDS-PAGE for the NMR sample indicates that while substantial AbpA remains monomeric, some larger multimeric species are formed. (**C**) Overlay of ^1^H-^15^N HSQC spectrum of AbpA alone (without the LPKTS motif) with that after the addition of equivalent amount of SrtB (red). No spectral changes are observed indicating the absence of an interaction.

To further examine the polymerization of AbpA, an NMR titration was performed with AbpA (either with the LPKTS motif present or absent) and SrtB. Upon immediate addition of SrtB, the specific signal in the spectrum of AbpA- LPKTS shows peak broadening indicating an interaction occurs (Fig. 10A). Furthermore, after two hours new signals appear, which are characteristic of short peptide fragments consistent with SrtB cleavage of the LPKTS motif (Fig. 10B). Furthermore, an overall decrease in signal intensity is seen suggestive of the presence of a higher order species (Fig. 10B). This is confirmed by the SDS-PAGE gel of the NMR sample in which the AbpA ladder is observed (Fig. 10B inset). No peak changes were observed using the construct of AbpA with the LPKTS motif absent, which is consistent with a lack of an interaction (Fig. 10C).

## Discussion

Colonization of the oral cavity by streptococci involves bacterial interactions with salivary components^37^,^38^. In this study, we have determined that *S. parasanguinis* AbpA and its homologs in oral streptococci belong to a unique class of bacterial proteins that bind to salivary amylase. This class shares a conserved N-terminal signal sequence domain and a C-terminal CWSS domain that is only found in oral streptococci. The C-terminal domain has an LPKTS motif followed by a very short AVK tail, and is essential for the surface display of AbpA and for the amylase binding. This short AVK tail is distinct from the longer tails recognized by canonical sortases, which normally includes both hydrophobic and charged features^3^. The long charged tails from canonical sortases play a role in retaining targeted proteins at the bacterial cell membrane for sortases to modify^39^,^40^,^41^. The short C-terminal tail of AbpA from *S. parasanguinis* and *S. gordonii* may have reduced its ability to retain AbpA, which could explain why AbpA is readily released into the culture supernatant.

The *abpA-srtB* gene organization is highly conserved in oral streptococci. Every *abpA*-like gene identified to date is followed by a *srtB* gene on the chromosome, which encodes an accessory sortase. N-terminal and C-terminal conserved motifs of AbpA are essential for recognition and modification by SrtB. SrtB of *S. aureus* and *S. pneumoniae* failed to complement the *srtB* mutant of *S. parasanguinis in vivo* or process the recombinant AbpA *in vitro*. Comparison of the oral streptococcal SrtB proteins with reported SrtA and SrtB proteins from other bacteria revealed that they share similar active sites, as they are required for the conserved sorting reactions. SrtB of *S. parasanguinis* indeed possesses the same activity (Fig. S1D). However, sequence conservation across the entire sortase open reading frame is very low, reflecting their diverse function. In addition, both N-terminal and C-terminal region are longer in oral streptococcal SrtB sortases and these structural features may contribute to the selective recognition of AbpA-like proteins.

*In vivo* and *in vitro* studies showed that AbpA could be processed into a ladder profile on the cell wall by oral streptococcal SrtB, but not by the housekeeping SrtA, or SrtB from either *S. pneumoniae* or *S. aureus*. The ladder profile was not evident in culture supernatants where only monomeric AbpA accumulates. This indicated that such presentation of AbpA may only occur on the cell wall, where the sortase B locates. As sortases are transmembrane proteins, it is possible that AbpA in the cultural supernatant is too distant from the enzyme so that the reaction could not be catalyzed. Interestingly, the *in vitro* protein ladder profile reconstituted with AbpA and SrtB is different from that observed in the cell wall fraction *in vivo*. The *in vitro* ladder profile may represent simple association of AbpA according to its molecular weight (Fig. 3B). However, in the *in vivo* study, additional protein bands smaller than the AbpA dimer were observed (Fig. 1B), which indicates that other unknown cellular factors may be involved in the process. In Gram-positive bacteria, many cell surface pili are assembled by different protein subunits such as the pilius-1 from *S. pneumoniae*, which includes one major component (RrgB) and two minor components (RrgA and RrgC)^42^. Further investigations on the composition of AbpA ladder pattern observed is ongoing, which should help reveal underlying mechanisms.

SrtB from *S. parasanguinis* has dual functions. It can catalyze the reaction to anchor AbpA to the cell wall first, and then process AbpA to form the ladder profile. This pattern of AbpA distribution is reminiscent of pili or fimbriae found on many Gram-positive bacteria^39^,^43^,^44^,^45^. However EM studies we conducted did not provide evidence indicative of a pilus-like structure for AbpA. Nevertheless, SrtB from *S. parasanguinis* can catalyze the reaction of anchoring AbpA to the cell wall first, and then process AbpA into the ladder pattern. Its role as a hybrid sortase is also supported by its significant sequence identity with both SrtC and SrtA sortase. The potential covalent linkage of AbpA into a ladder pattern would offer functional advantages over the presentation of a simple, cell wall anchored monomeric entity. Such a presentation pattern not only offers a degree of mechanical strength, but would enable to the adhesive properties of AbpA to engage optimally with its host binding partner, human α-amylase. AbpA may also function as a regulator as mutation of *abpA* in *S. gordonii* down-regulates expression of a bacterial two component signal transduction system and a variety of metabolic pathways^46^. Abundant distribution of AbpA on the cell surface would assist its role as a receptor and amply signals it transduces.

In addition, we solved the solution structure of AbpA by NMR and SAXS, which revealed a distinctive two-domain structure comprising an N-terminal extended helical coiled coil and a globular mixed α/β domain at C-terminus adjacent to the sortase signal motif. The overall shape of AbpA resembles a boomerang shape and the key residues that mediate the binding to human salivary amylase line the helical coiled-coil feature. NMR evidence is also provided to support that the C-terminal domain interacts with SrtB. Our NMR structure of AbpA reveals an architectural resemblance with the fibrillary coiled-coil structure of the A_3_VP_1_ fragment of *S. mutans* AgI/II. These data together with the presence of a C-terminal domain harboring the SrtB motif support its role in anchoring AbpA to the bacterial cell surface and possible the assembly of AbpA into a ladder profile on the cell surface. In this scenario the helical coiled-coil domain would likely project away from the anchored C-terminus for optimal presentation and interaction with its receptor.

The present studies uncovered a novel mechanism by which a single sortase SrtB of *S. parasanguinis* has the dual role in anchoring AbpA to the cell surface, and processing AbpA. Our data support SrtB as a distinct class of sortases that target an unusual cell wall sorting signaling within the C-terminus of its cognate substrate AbpA. This unique AbpA-SrtB pair is highly conserved in oral streptococci that have adapted to grow and survive in the oral cavity rich in salivary amylase. Our studies illustrate this unique bacterium-host interaction.

## Methods

### Construction of *abpA* mutant and complementation strains in *S. parasanguinis*

Allelic replacement mutagenesis strategy was used to construct *abpA* mutants. All strains and primers used in this study are listed in SI Tables 1 and 2. A 2491-bp PCR fragment of *abpA* was amplified from *S. parasanguinis* using the primer pair, abpA-F1/abpA-R1, and then cloned into the pGEM-T easy vector (Promega). The resulting construct was used as template, inverse PCR were performed with the primer pair, abpA-F2/abpA-R2, in which *Eco*RI was introduced. The resulting PCR product was digested with *Eco*RI and ligated in-frame with a same enzyme digested promoterless kanamycin resistance cassette *aphA3*^47^ to generate the plasmid pAL820. Through the inverse PCR, a 480-bp DNA fragment that encodes the 23–183 amino acid residues of AbpA was deleted and replaced with *aphA3*. This plasmid was used to transform *S. parasanguinis* FW213 and the kanamycin resistance transformants were isolated. The replacement of the genomic copy of *abpA* with the disrupted alleles by double-crossover recombination in the strain AL821 was confirmed by colony PCR and sequencing analysis.

To construct the *abpA* complement strain, the full-length *abpA* gene was PCR amplified from the genomic DNA of *S. parasanguinis*, using the primer pair, abpA-F3/abpA-R3, with engineered *Sal*I and *Kpn*I restriction enzyme sites. The PCR product was digested with *Sal*I and *Kpn*I, and cloned into pVPT-gfp^48^ to generate pAL822. This plasmid was transformed into the *abpA* mutant AL821 to construct the complemented strain AL823.

### Expression and purification of recombinant AbpA and SrtB

*S. parasanguinis abpA* was amplified with the primer pair, abpA-F4/abpA-R4, in which *Nco*I and *Xho*I were introduced. PCR product was digested with *Nco*I and *Xho*I and ligated with the same enzyme digested pET28a-sumo to generate plasmid pAL832. A recombinant SrtB strain was also constructed. In brief, *srtB* was amplified with the primer pair, srtB-F5/srtB-R5, in which *Bam*HI and *Xho*I were introduced. The PCR product was digested with *Bam*HI and *Xho*I and ligated with the same enzyme digested pET28a-sumo to produce plasmid pAL833. These two plasmids were transformed into *E. coli* BLR (DE3) to generate recombinant AL832 and AL833 respectively for protein expression and purification. Recombinant AbpA and SrtB were induced and purified as described^49^. The recombinant AbpA has a C-terminal His-tag and the SrtB has an N-terminal His-SUMO-tag. Same strategy was applied to construct, express and purify SrtB-His, His-SUMO-AbpA (24-138aa) and other AbpA variants. Site-directed mutagenesis was used to construct AbpA variants by PCR using a QuikChange mutagenesis kit (Stratagene).

### Subcellular localization of AbpA

Proteins from different subcellular fractions were prepared by the method described previously^50^. Briefly, 10 ml of exponentially grown *S. parasanguinis* or *S. gordonii* cells were harvested, washed and subjected to cell lysis in 200 μl of spheroplasting buffer as reported^50^ with 60 U mutanolysin (Sigma). The supernatant separated from the spheroplast by centrifugation was used as the cell wall-associated protein fraction. The pellet re-suspended in 200 μl of spheroplasting buffer was used as the cytoplasmic protein fraction.

### Amylase-binding assays

Human saliva was used as the source of amylase. 3 ml exponentially grown *S. parasanguinis* or *S. gordonii* cells were harvested, and washed with PBS buffer once. The cells were resuspended with 150 μl saliva and incubated 1 h at 4 °C. Cells and the saliva were separated by centrifugation (8000 × g rpm for 5 min) and the cell pellets were washed three times with PBS to remove the residual saliva. The cell pellets and the saliva supernatant were boiled in SDS-PAGE loading buffer and subjected to SDS-PAGE or Western blot analysis.

### Immunoelectron microscopy

One ml of bacterial cells were washed twice with PBS and suspended in 500 μl PBS. A drop of bacterial suspension was placed onto glow-discharged carbon-only grids (Electron Microscopy Sciences), and fixed with 0.1% glutaraldehyde. After washing in 10 mM glycine buffer, the grids were blocked for 1 h in PBS with 1% BSA. Cells were incubated with a primary antibody diluted 1:200 in PBS with 1% BSA for 1 h, followed by washing. Grids were incubated with gold-labeled goat anti-rabbit IgG (Electron Microscopy Sciences) diluted 1:20 in PBS with 1% BSA for 1 h, followed by washing in PBS with 1% BSA. The grids were washed five times with water before they were stained with 1% phosphotungstic acid. Samples were analyzed using FEI Tecnai T12 electron microscope.

### NMR spectroscopy and structure calculation

NMR samples were prepared as described previously^49^. NMR spectra were collected at 310 K on Bruker DRX600 and DRX800 spectrometers equipped with cryo-probes. NMR structural determination including the aliphatic and aromatic side chain H and C assignments, the backbone assignment, and the assignment of high content of alanine residues was carried out as described^49^.

The ARIA protocol was used for completion of the NOE assignment and structure calculation^51^. Dihedral angle restraints derived from TALOS were also incorporated in the calculation. The frequency window tolerances for assigning NOEs were ±0.05 ppm for direct proton dimensions, ±0.05 ppm for indirect proton dimensions, and ±0.5 ppm for nitrogen dimensions and ±1.1 ppm for carbon dimensions. The 20 lowest energy structures had no NOE violations >0.5 Å and no dihedral angle violations >5°. Residual Dipolar Couplings (RDCs) were measured using In-phase Anti-phase (IPAP) experiments with samples containing 14 mg ml^−1^ Pf1 phage. The alignment tensor was obtained from PALES and then used in MODULE^52^ for refinement of the relative domain orientation. A set of ^1^H-^15^N RDCs was used to refine the relative orientation of the two domains within the SAXS density, using the program MODULE^52^.

### Amylase, SrtB and AbpA NMR titration

In amylase-binding experiments, unlabeled amylase was added to ^15^N labeled AbpA according to stoichiometric ratio to perform NMR titration. Maximal five-fold amylase was added to AbpA in order to reach saturation. In SrtB/AbpA titration, equivalent amount of unlabeled SrtB was mixed with ^15^N labeled AbpA, and spectra were recorded at different time points.

### Small angle X-ray scattering

SAXS samples were prepared using BIOSAXS robot in 96-well plate. Exposures (20 s) in triplicate were collected on protein at 3 mg ml^−1^ passed through a flow capillary. I(0) and the pair distance distribution function P(r) were calculated using software SCATTER^53^. Scattering from sample buffer 50 mM NaCl, 100 mM KH_2_PO_4_, pH 5.7 was subtracted. Guinier analysis showed no signs of radiation damage or aggregation. Twenty low-resolution ab initio models from GASBOR^54^ were automatically averaged using DAMAVER.

## Additional Information

**How to cite this article**: Liang, X. *et al*. A distinct sortase SrtB anchors and processes a streptococcal adhesin AbpA with a novel structural property. *Sci. Rep.* **6**, 30966; doi: 10.1038/srep30966 (2016).

## Supplementary Material

Supplementary Information

## Figures and Tables

**Table 1:** NMR structure calculation statistics data for *S. parasanguinis* AbpA.

	AbpA (pdb code: 2ND4)
**NMR distance & dihedral constraints**	
Distance constraints	
Total NOE	1671
Intra-residue	690
Inter-residue	981
Sequential (|i-j| = 1)	443
Medium-range (|i-j| < 4)	93
Long-range (|i-j > 5)	345
Intermolecular	0
Hydrogen bonds	0
Total dihedral angle restraints	
Phi	136
Psi	136
Total RDCs	95
**Structure Statistics**	
Violations (mean and s.d.)	
Distance constraints (Å)	0.035 ± 0.004
Dihedral angle constraints (°)	1.40
Max. dihedral angle violation (°)	1.11
Max. distance constraint violation (Å)	0.46
Deviations from idealized geometry	
Bond lengths (Å)	0.004 ± 0.001
Bond angles (°)	0.56 ± 0.001
Impropers (°)	1.645 ± 0.076
Average pairwise r.m.s.d. 10 (Å)	
Heavy	0.323
Backbone	0.349

**Table 2:** SAXS data collection, processing and modelling for AbpA.

*P(r)* function calculation	pH 5.0
*q*-range (Å^−1^)	0.022–0.219
*R*_g_ (Å)	26.4 ± 0.01
*I*(0)	90 ± 0.06
*D*_max_ (Å)	102
Estimated molecular mass^a^	23 kDa
Mass calculated from sequence	19 kDa
*Ab initio* GASBOR modelling	
Ensemble average *χ*^2^ to raw data	1.98 ± 0.02
NSD^b^	1.13 ± 0.06

^a^By normalisation against data for BSA, calculated using the formula [I(0)_FAP_ ÷ I(0)_BSA_*66 kDa] where I(0)_BSA_ was 188.3.

^b^For the definition of normalised spatial discrepancy (NSD), see Kozin & Svergun, 2001).
