# Supplementary material for: A distinct sortase SrtB anchors and processes a streptococcal adhesin AbpA with a novel structural property
Source: Sci Rep. 2016 Aug 5;6:30966. doi: 10.1038/srep30966 (PMC4974636; doi:10.1038/srep30966)
Supplement: Supplementary Information [file srep30966-s1.pdf]

Supplementary Information

A distinct sortase SrtB anchors and processes a streptococcal adhesin AbpA with a novel structural property

Xiaobo Liang[a,1,2], Bing Liu[b,1], Fan Zhu[a,1], Frank A. Scannapieco[c], Elaine M Haase[c], Steve Matthews[b*] and Hui Wu[a*]

[a]Departments of Pediatric Dentistry and Microbiology, University of Alabama at Birmingham School of Dentistry, Birmingham, AL , USA; [b]Department of Life Sciences, Imperial College London, London, UK; [c]Department of Oral Biology, University at Buffalo, Buffalo, NY, USA

Key words: sortase, amylase binding protein A, LPXTG motif, streptococcus

Running Title: New sortase and adhesin substrate system

[1] These three authors contribute equally to the manuscript

[2] Present Address: Yunnan Institute of Food Safety, Kunming University of Science and Technology, Kunming, Yunnan, China.

[*] To whom correspondence should be addressed. E-mail: hwu@uab.edu or s.j.matthews@imperial.ac.uk

Methods

Bacterial strains, plasmids and growth conditions

The bacterial strains and plasmids used in this study are listed in SI Table 1. *Escherichia coli*, *S. parasanguinis* and *S. gordonii* strains were cultured in the growth conditions described previously[1]. For bacterial growth, 100 μg ml^{-1} ampicillin, 50 μg ml^{-1} kanamycin or 300 μg ml^{-1} erythromycin were used in *E. coli*, 125 μg ml^{-1} kanamycin or 5 μg ml^{-1} erythromycin were used in *S. parasanguinis*, 1 mg ml^{-1} kanamycin or 5 μg ml^{-1} erythromycin were used in *S. gordonii*.

Molecular cloning techniques

E. coli plasmid DNA was isolated using the mini-prep DNA preparation kit (Qiagen). The genomic DNA of *S. parasanguinis* and *S. gordonii* were extracted using the Puregene DNA isolation kit (Gentra System). Polymerase chain reaction (PCR) was performed with KOD hot start DNA polymerase (Novagen), using the GeneAmp PCR system 9700 (PE Applied Biosystems). Primers used for the amplification of DNA fragments are listed in SI Table 2. Restriction enzymes and T4 DNA ligase (New England Biolabs) were used according to the manufacturer's instructions. Competent *E. coli* cells were prepared and transformed by standard techniques [2]. *S. parasanguinis* was transformed by electroporation using a gene pulser (Bio-Rad Laboratories) as described previously[3]. *S. gordonii* was transformed by natural transformation method [4].

Construction of srtA and srtB mutants and complementation strains in *S. parasanguinis*

The allelic replacement strategy was used in *srtA* and *srtB* mutagenesis. *srtA* and *srtB* and their flanking regains were amplified form *S. parasanguinis* FW213 genomic DNA by PCR with primer pairs srtA-F1/srtA-R1 and srtB-F1/srtB-R1, respectively. PCR products were cloned into *Hin*dIII and *Xho*I digested and end-blunted pSU21[7], yielding pBYL12 (containing *srtA*) and pBYL11 (containing *srtB*) respectively. Inverse PCR with two primer pairs of srtA-F2/srtA-R2 and srtB-F2/srtB-R2, in each of which the *Bgl*II and *Bam*HI enzyme sites were introduced. The resulting DNA fragments were digested with *Bgl*II or *Bam*HI and ligated with the *Bam*HI digested erythromycin resistance gene fragment to generated pBYL16 and pBYL17, respectively. These plasmids were transformed into *S. parasanguinis* FW213 and the erythromycin resistance transformants BYL32 and BYL31 were isolated, confirmed by colony PCR and were used in the study.

The *srtB* complement strain was constructed using plasmid pDL276. Briefly, the full length *srtB* gene was amplified from *S. parasanguinis* using the primer pair srtB-F3/srtB-R3, in which *Sac*I and *Sph*I were introduced. The PCR product was digested with *Sac*I and *Sph*I and was inserted into same enzymes digested pDL276, yielding plasmid pBYL37. This plasmid was transformed into BYL31 and the colonies resistant to both kanamycin and erythromycin were confirmed and named as BYL37.

Construction of srtB mutant and complementation strains in *S. gordonii*

A 2202-bp PCR fragment of *srtB* was amplified from *S. gordonii* using the primer pair, srtB-F1g/srtB-R1g, and then cloned into the pGEM-T easy vector. The resulting construct was used as template, inverse PCR were performed with the primer pair srtB-F2g/srtB-R2g, in which *Hin*dIII site was introduced. The resulting PCR product was digested with *Hin*dIII and ligated in-frame with a same enzyme digested promoterless kanamycin resistance cassette *aphA3* to generate the plasmid pAL824. Through the inverse PCR, a 531-bp DNA

fragment which coding the 16-193 amino acids of SrtB protein was deleted. This plasmid was used to transform *S. gordonii* and the kanamycin resistance transformants were isolated, confirmed by PCR and sequence analyses and was named as AL825.

The complement strain was constructed using pVPT-gfp vector. *srtB* gene was PCR amplified from the genomic DNA of *S. gordonii*, using the primer pair srtB-F3g/srtB-R3g with engineered *Sal*I and *Bam*HI restriction enzyme sites. The PCR product was digested with *Sal*I and *Bam*HI, and cloned into pVPT-gfp [8] to generate pAL826. This plasmid was transformed into the *abpA* mutant AL825 to construct complemented strains AL827.

Cross-complementation of srtB mutation between *S. parasanguinis and S. gordonii*

To test if the SrtB from *S. parasanguinis* is functional in *S. gordonii* and vice versa, two cross-complementation strains were constructed. The *S. gordonii srtB* was amplified with the primer pair srtB-F4g/srtB-R4g and was cloned into pDL276. The resulting plasmid (pAL828) was transformed into *S. parasanguinis* mutant BYL31 to generate AL829. Similarly, the *S. parasanguinis srtB* was amplified with the primer pair srtB-F4/srtB-R4 and was cloned into pVPT-gfp. The resulting plasmid (pAL830) was transformed into *S. gordonii* mutant AL825 to generate AL831. AL829 and AL831 were used in amylase binding assays.

Functional study of SrtB of *S. aureus* and *S. pneumoniae*

For the in vivo studies, two srtB cross complementation strains were constructed. Briefly, srtB was amplified by PCR from *S. aureus* COL and *S. pneumoniae* Tigr4 genome respectively using the primer pairs of srtB-F1c/srtB-R1c and srtB-F1t/srtB-R1t. The PCR products were digested with *Sph*I/*Eco*RI and ligated with pDL276. The resulting plasmids (pAL829 and

pAL831) were transformed into *S. parasanguinis srt*B mutant to generatetwo cross-complementation strains, AL838 and AL839. The strains were used in amylase binding assays.

For the in vitro studies, two recombinant SrtB proteins were expressed in *E. coli*. Primer pair of srtB-F2c/srtB-R2c and srtB-F2t/srtB-R2t was used in PCR to amplify srtB from *S. aureus* COL and *S. pneumoniae* Tigr4 genome respectively. The PCR product was digested with *Bam*HI/SacI and ligated with pET28a-sumo, and the resulting plasmids were transformed into *E.coli* BLR. Recombinant SrtB were expressed, purified and used in the enzymatic assay with the recombinant AbpA as a substrate.

Construction of abpA deletion mutants and site-directed mutants in *S. parasanguinis*

Using abpA-F3 as a forward primer, in combination with different reverse primers, deletion and site-directed mutation alleles for the conserved C-terminal cell wall sorting signal motif of AbpA were generated by PCR amplification. The primers used are listed in SI Table2. The PCR products were digested with *Sal*I and *Kpn*I, ligated with same enzymes digested pVPT-gfp, yielding the mutation plasmids. These plasmids were then transformed into the *abpA* mutant strain AL821. The resulting strains were used in amylase binding assays.

Preparation of polyclonal antibody against AbpA

1.0 mg of the purified AbpA of *S. parasanguinis* was used to generate polyclonal antisera in rabbit by Cocalico Biologicals, Inc (Reamstown, PA, USA). The titer and the specificity of the antiserum to AbpA were tested by ELISA and western blot analysis, respectively.

Construction of AbpA-GFP swap strains in *S. parasanguinis*

Using pAL822 as a template, we performed inverse PCR with a forward primer abpA-F and three different abpA-R primers (abpA-R1, -R2 and –R3 designed for swap1, 2 and 3 constuct respectively), in which the *Sph*I and *Xho*I sites were introduced. At the same time *gfp* was amplified from pVPT-gfp with the primer pair gfp-F/gfp-R which also contains *Sph*I and *Xho*I sites. Both PCR products were digested with *Sph*I and *Xho*I and ligated together to produce plasmid pAL834 and its variants, in which the *gfp* gene was inserted between the coding sequence of the N-terminal signal peptide and the C-terminal cell wall anchor motif of AbpA. These plasmids were transformed into *S. parasanguinis* to obtain AbpA-GFP swap strains, Swap1, Swap2 and Swap3. Another strain (SP-GFP) in which GFP was fused only with the N-terminal conserved domain of AbpA. This stain was constructed similarly and used as a control.

In vitro enzyme activity assay of SrtB

Recombinant AbpA, AbpA-His or AbpA-Gfp and SrtB or SrtB-His were incubated together in 37°C for 1 h and then probed with anti-AbpA or anti-His to detect cleavage during the reaction. The resulting AbpA peptides were identified with Mass Spectrometric analyses and N-terminal amino acid sequencing (University of Texas Medical Branch Biomolecular Resource Facility).

SDS-PAGE and Western blot analysis

Protein samples were separated on 10% SDS-polyacrylamide gel and then stained with Coomassie blue staining R-250. For Western blot analysis, the separated proteins were transferred to nitrocellulose membranes. The membranes were blocked by 5% skimmed milk in PBS for 1 h prior to probing

with AbpA and other antibodies diluted at 1:2000 in PBS with 0.1% Tween-20. Horseradish peroxidase-conjugated anti-rabbit and ECL Western blot detection reagents (GE Healthcare) were used to detect AbpA and other proteins.

Figure Legends

Supplementary Figure S1. Conservation and uniqueness of AbpA. **A.** Comparison of *abpA-srtB* gene locus among different oral streptococcal species. **B.** Alignment of deduced protein sequence of AbpA homologs. The N-terminal and C-terminal conserved motifs were marked by black rectangle. Secondary structure is shown above the alignment; β-strands (β1-3) are colored as yellow arrows and the five helices (α1-5) as red rectangles. **C.** Schematic representation of typical CWSS and C terminus of AbpA. +, positively charged tail.

Supplementary Figure S2. NMR titration of AbpA with amylase. **A**. ^{1}H-^{15}N HSQC spectra of *S. parasanguinis* AbpA collected in the absence (left) and presence (middle - 0.5 molar ration and right - 1 molar ratio) of human α-amylase. **B.** Amylase titration for mutant AbpA variants. ^{1}H-^{15}N HSQC spectra of *S. parasanguinis* AbpA mutant proteins collected in the absence (black) and presence (red) of a 5-fold excess of human α-amylase. From left to right: KK37/38A, YY132/133A and VL117/118A.

Supplementary Table S1. Bacterial strains and plasmids used in this study.

Strain or plasmid	Relevant characteristics	Reference or source
Strains		
E. coli		
Top10	F- *mcrA* Δ(*mrr-hsdRMS-mcrBC*) φ80*lacZ*ΔM15 Δ*lacX*74 *nupG recA1 araD*139 Δ(*ara-leu*)7697 *galE*15 *galK*16 *rpsL*(Str^r) *endA1* λ$^-$	Invitrogen
BLR(DE3)	F$^-$ *ompT hsdS* $_B$(r$_B^-$ m$_B^-$) *gal dcm* (DE3) Δ(*srl – recA*)*306*::Tn*10* (Tet^r).	Novagen
BL21(DE3)	*huA2 [lon] ompT gal (λ DE3) [dcm] ΔhsdS λ DE3 = λ sBamHIo ΔEcoRI-B int::(lacI::PlacUV5::T7 gene1) i21 Δnin5*	NEB
AL832	BLR(DE3) with pAL832 plasmid; Kan^r	This study
AL833	BLR(DE3) with pAL833 plasmid; Kan^r	This study
AL840	BL21(DE3) with pAL840 plasmid; Kan^r	[9]
AL841	BL21(DE3) with pAL841 plasmid; Kan^r	This study
AL842	BL21(DE3) with pAL842 plasmid; Kan^r	This study
AL843	BL21(DE3) with pAL843 plasmid; Kan^r	This study
AL844	BL21(DE3) with pAL844 plasmid; Kan^r	This study
AL845	BL21(DE3) with pAL845 plasmid; Kan^r	This study
AL846	BL21(DE3) with pAL846 plasmid; Kan^r	This study
S. parasanguinis		
FW213	*S. parasanguinis* parent strain	[10]
AL821	FW213 *abpA*::*aphA3*; Kan^r	This study
AL823	FW213 *abpA*::*aphA3*::pAL822; Kan^r, Em^r	This study
BYL31	FW213 *srtB*::*emr*; Em^r	This study
BYL32	FW213 *srtA*::*emr*; Em^r	This study
BYL37	FW213 *srtB*::*emr*::pBYL37; Em^r, Kan^r	This study
AL829	FW213 *srtB*::*emr*::pAL828; Em^r, Kan^r	This study
AL835	FW213::pAL834; Em^r	This study
AL837	FW213::pAL836: Em^r	This study
AL838	FW213 *srtB*::*emr*::pAL829; Em^r, Kan^r	This study
AL839	FW213 *srtB*::*emr*::pAL831; Em^r, Kan^r	This study
S. gordonii		
Challis	*S. gordonii* parent strain	[11]

AL825	Challis srtB:: *aphA3*; Kan^r	This study
AL827	Challis srtB:: *aphA3*::pAL826; Kan^r, Em^r	This study
AL831	Challis srtB:: *aphA3*::pAL830; Kan^r, Em^r	This study
Plasmids		
pGEM-T	Commercial TA cloning vector	Promega
pVPT-gfp	*E. coli* & *Streptococcus* shuttle vector	[8]
pDL276	*E. coli* & *Streptococcus* shuttle vector	[12]
pET28a-sumo	*E.coli* expression vector with a SUMO tag	[13]
pSU21	*E. coli* cloning vector	[7]
pAL820	pGEM-*abpA-aphA3*	This study
pAL822	pVPT-*abpA*	This study
pBYL16	pSU21-*srtA-emr*	This study
pBYL17	pSU21-*srtB-emr*	This study
pBYL37	pDL276-*srtB*	This study
pAL824	pGEM-*srtB(g)* [a]-*aphA3*	This study
pAL826	pVPT-*srtB(g)*	This study
pAL828	pDL276-*srtB(g)*	This study
pAL829	pDL276-*srtB(c)*	This study
pAL830	pVPT-*srtB*	This study
pAL831	pDL276-*srtB(t)*	This study
pAL832	pET28a-*abpA*	This study
pAL833	pET28a-sumo-*srtB*	This study
pAL834	pVPT-*abpA-gfp*	This study
pAL840	pET28a-sumo-*abpA∆C10aa*	[9]
pAL841	pET28a-sumo-*abpA(24-138aa)*	This study
pAL842	pET28a-sumo-*abpA(139-207aa)*	This study
pAL843	pET28a-sumo-*abpA-KK37/38A*	This study
pAL844	pET28a-sumo-*abpA-YY132/133A*	This study
pAL845	pET28a-sumo-*abpA-VL117/118A*	This study
pAL846	pET28a-*srtB*	This study

[a]g means this gene comes from *S. gordoni* CH1, c means this gene comes from *S. aureus* Col, t means this gene comes from *S. pneumonia Tigr4*

Supplementary Table S2. Primers used in this study

Primer	Sequence[b]
abpA-F1	GGGCACGTAAGTTAGCTGAGTT
abpA-R1	TGAATCAGGGATTTGACGAAGATAAAGGT
abpA-F2	CCTGAATTCCAAATACAGGTGCTGCTGCAGCAAATACTGCAAGAGCTG
abpA-R2	CTCGAATTCGTTGACAAAGACGGTAAAACAG
abpA-F3	ATCGTCGACATGAAAAAAGTTTTATTATCATCAGTAGC
abpA-R3	CATGGTACCAAATTATTTAACTGCGCTTGTTTTTGGA
abpA-F4	GCACCATGGCTATGAAAAAAGTTTTATTATCATCAGTAG
abpA-R4	ATACTCGAGTTTAACTGCGCTTGTT
abpA-del1	CATGGTACCAAATTAACCAGCTTTAGCATCAGCC
abpA-del2	CATGGTACCAAATTAAGCTTTACCAGCTTTAGC
abpA-del3	CATGGTACCAAATTAGCTTGTTTTTGGAAGAGC
abpA-L/N	CATGGTACCAAATTATTTAACTGCGCTTGTTTTTGGATTAGCTTTACCAG
abpA-L/R	CATGGTACCAAATTATTTAACTGCGCTTGTTTTTGGACGAGCTTTACCAG
abpA-P/A	CATGGTACCAAATTATTTAACTGCGCTTGTTTTAGCAAGAGCTTTACCAG
abpA-P/D	CATGGTACCAAATTATTTAACTGCGCTTGTTTCATCAAGAGCTTTACCAG
abpA-K/E	CATGGTACCAAATTATTTAACTGCGCTTGTTTCTGGAAGAGCTTTACCAG
abpA-K/L	CATGGTACCAAATTATTTAACTGCGCTTGTTAGTGGAAGAGCTTTACCAG
abpA-T/A	CATGGTACCAAATTATTTAACTGCGCTTGCTTCTGGAAGAGCTTTACCAG
abpA-T/S	CATGGTACCAAATTATTTAACTGCGCTTGATTTTGGAAGAGCTTTACCAG
abpA-S/W	CATGGTACCAAATTATTTAACTGCCCATGTTTTTGGAAGAGCTTTAC
abpA-S/G	CATGGTACCAAATTATTTAACTGCCCCTGTTTTTGGAAGAGCTTTAC
abpA-S/R	CATGGTACCAAATTATTTAACTGCCCTTGTTTTTGGAAGAGCTTTAC
abpA-K37/38A F	CTGCTGCATCACGCCATGAAACATATGCCGCTTGGATCAATTGGTTAGATGCACTTG
abpA-K37/38A R	CAAGTGCATCTAACCAATTGATCCAAGCGGCATATGTTTCATGGCGTGATGCAGCAG
abpA-H56AF	CTTCTTTGATTTCTGCTTCAGCAGCTGCTACTTGAGTGTTAGCTT
abpA-H56AR	AAGCTAACACTCAAGTAGCAGCTGCTGAAGCAGAAATCAAAGAAG
abpA-Y114A F	ATTTTTGTTGAAGAACTTGGATGGCACGGTTGCGAACTGTGTTGTATG
abpA-Y114A R	CATACAACACAGTTCGCAACCGTGCCATCCAAGTTCTTCAACAAAAAT
abpA-VL117/118AA F	GCAGCTTCAATGTATTTTTGTTGAGCAGCTTGGATGTAACGGTTGCGAACTG
abpA-VL117/118AAR	CAGTTCGCAACCGTTACATCCAAGCTGCTCAACAAAAATACATTGAAGCTGC
abpA-Y132/133AF	TTAGCTTCTACAGCTGTTTCATCGGCGGCGTTACCTTGAGCTTTAGCAGCTTC
abpA-Y132/133AR	GAAGCTGCTAAAGCTCAAGGTAACGCCGCCGATGAAACAGCTGTAGAAGCTAA
abpA-V138A F	CATTTGTACGGTTAGCTTCTGCAGCTGTTTCATCGTAGTAG
abpA-V138A R	CTACTACGATGAAACAGCTGCAGAAGCTAACCGTACAAATG
AbpA-138aaR	ATGCCTCGAG TACAGCTGTTTCATCGTAGTAG
AbpA-24aaF	ATGCGGATCCCAAGGTGAAAACCCAAGTG

AbpA-139aaF	ATGC GGATCCGAAGCTAACCGTACAAATGAA
abpA-207aaR	GACACCTCGAGTTATTTAACTGCGCTTG
srtA-F1	GTCAAACGGACCAATGTAG
srtA-R1	CGTACATAATTTCCTCCTCAC
srtB-F1	GCACCTGTATTTGCACAAGG
srtB-R1	CTTCGTTTGCCCATCTTCG
srtA-F2	TGGGGGAGATCTATCTTCTAGCACAGCT
srtA-R2	TTTCTCAGCAGATCTCATTTCATTGAT
srtB-F2	CTCGACGGATCCCGTCATCAATGCCC
srtB-R2	GCAACGATGTTGAGGATCCTATTGCGTAAAC
srtB-F3	TTTGAGCTCTAATTAGCTCGAAACGAATATCA
srtB-R3	TTAGCATGCATTTCCTACAAATACTAGACATA
srtB-F4	ATACGTCGACATGAAAAGAGCTGAGA
srtB-R4	CTAAGGATCCTTGTTGACCACGTGTT
srtB-F5	ATCGGATCCATGAAAAGAGCTGAGA
srtB-R5	AATCTCGAGTTATTATTGTTGACCA
srtB-F1g c	AGTTTTATTGTCAAGCGTG
srtA-R1g	ATAAACAATCCAGCGC
srtB-F2g	ATCGGATCCTCTTATTTGGAGCCTT
srtB-R2g	CATGGATCCATGGGCTTGGTTATTG
srtB-F3g	ACCGTCGACATGAGTCAAAATGCTA
srtB-R3g	TCAGGGGATCCCTATCTAGTAGGAGTATAT
srtB-F4g	AGTGCATGCGATGAGTCAAAATGCTA
srtB-R4g	TCTGAATTCCTATCTAGTAGGAGTA
srtB -F1c	ATTGCATGCAGATGAGAATGAAGCGATTTTT
srtB-R1c	ACTGAATTCCTAACTTACCTTAATTATTTTTG
srtB -F2c	AGTGGATCCATGAGAATGAAGCGATTTTT
srtB-R2c	GATGAGCTCTTATTAACTTACCTTAATTATTTTTG
srtB-F1t	ATTGCATGCAGATGGCGGTAATGGCGTATCC
srtB-R1t	ACTGAATTCCTACTGTTGTCCATCCTCCACCT
srtB-F2t	AGTGGATCCATGGCGGTAATGGCGTATCC
srtB-R2t	GATGAGCTCTTATTACTGTTGTCCATCCTCCACCT

[a] Restriction sites are underlined.

[b] g stands for *S. gordoni* CH1, c for *S. aureus* COL, t for *S. pneumoniae* Tigr4

Supplementary Table S3.

Summary of mutagenesis at the C-terminal domain of AbpA.

**Mutations**	**Sequences**	**Description**	**Amylase binding**
WT	KAGKALPKTSAVK		**+**
Del1(205-207)	KAGKALPKTS		**-**
Del2(200-207)	KAGKA		**-**
Del3(195-207)	-		**-**
200L/N	KAGKANPKTSAVK	Hydrophobic/uncharged	**-**
200L/R	KAGKARPKTSAVK	Hydrophobic/Hydrophilic	**-**
201P/A	KAGKALAKTSAVK	Hydrophobic/Hydrophobic	**-**
201P/D	KAGKALDKTSAVK	Hydrophobic/Hydrophilic	**-**
202K/E	KAGKALPETSAVK	Hydrophilic/Hydrophilic	**+**
202K/L	KAGKALPLTSAVK	Hydrophilic/Hydrophobic	**-**
203T/A	KAGKALPKASAVK	Uncharged/Hydrophobic	**-**
203T/S	KAGKALPKSSAVK	Uncharged/Uncharged	**-**
204S/W	KAGKALPKTWAVK	Uncharged/Hydrophobic	**+**
204S/G	KAGKALPKTGAVK	Uncharged/Hydrophobic	**+**
204S/R	KAGKALPKTRAVK	Uncharged/Hydrophilic	**+**

References

1 Wu, H., Zeng, M. & Fives-Taylor, P. The glycan moieties and the N-terminal polypeptide backbone of a fimbria-associated adhesin, Fap1, play distinct roles in the biofilm development of Streptococcus parasanguinis. *Infect Immun* **75**, 2181-2188 (2007).

2 J. Sambrook, E.F. Fritsch & T. Maniatis. *Molecular cloning: a laboratory manual*. Vol. 2 (Cold Spring Harbor Laboratory Press, 1989).

3 Fenno, J. C., Shaikh, A. & Fives-Taylor, P. Characterization of allelic replacement in Streptococcus parasanguis: transformation and homologous recombination in a 'nontransformable' streptococcus. *Gene* **130**, 81-90 (1993).

4 Petersen, F. C. & Scheie, A. A. Natural transformation of oral streptococci. *Methods Mol Biol* **666**, 167-180 (2010).

5 Kremer, B. H. *et al.* Characterization of the sat operon in Streptococcus mutans: evidence for a role of Ffh in acid tolerance. *Journal of bacteriology* **183**, 2543-2552, doi:10.1128/jb.183.8.2543-2552.2001 (2001).

6 Zhou, M., Fives-Taylor, P. & Wu, H. The utility of affinity-tags for detection of a streptococcal protein from a variety of streptococcal species. *Journal of microbiological methods* **72**, 249-256, doi:10.1016/j.mimet.2007.12.002 (2008).

7 Bartolome, B., Jubete, Y., Martinez, E. & de la Cruz, F. Construction and properties of a family of pACYC184-derived cloning vectors compatible with pBR322 and its derivatives. *Gene* **102**, 75-78 (1991).

8 Zhou, M., Fives-Taylor, P. & Wu, H. The utility of affinity-tags for detection of a streptococcal protein from a variety of streptococcal species. *J Microbiol Methods* **72**, 249-256 (2008).

9 Liu, B., Zhu, F., Wu, H. & Matthews, S. NMR assignment of the amylase-binding protein A from Streptococcus parasanguinis. *Biomolecular NMR assignments* **9**, 173-175, doi:10.1007/s12104-014-9568-9 (2015).

10 Cole, R. M., Calandra, G. B., Huff, E. & Nugent, K. M. Attributes of potential utility in differentiating among "group H" streptococci or Streptococcus sanguis. *J Dent Res* **55**, A142-153 (1976).

11 Rogers, J. D. *et al.* Identification and analysis of a gene (abpA) encoding a major amylase-binding protein in Streptococcus gordonii. *Microbiology* **144 (Pt 5)**, 1223-1233 (1998).

12 Dunny, G. M., Lee, L. N. & LeBlanc, D. J. Improved electroporation and cloning vector system for gram-positive bacteria. *Appl Environ Microbiol* **57**, 1194-1201 (1991).

13 Zhu, F. *et al.* Structural and functional analysis of a new subfamily of glycosyltransferases required for glycosylation of serine-rich streptococcal adhesins. *The Journal of biological chemistry* **286**, 27048-27057, doi:10.1074/jbc.M110.208629 (2011).

A

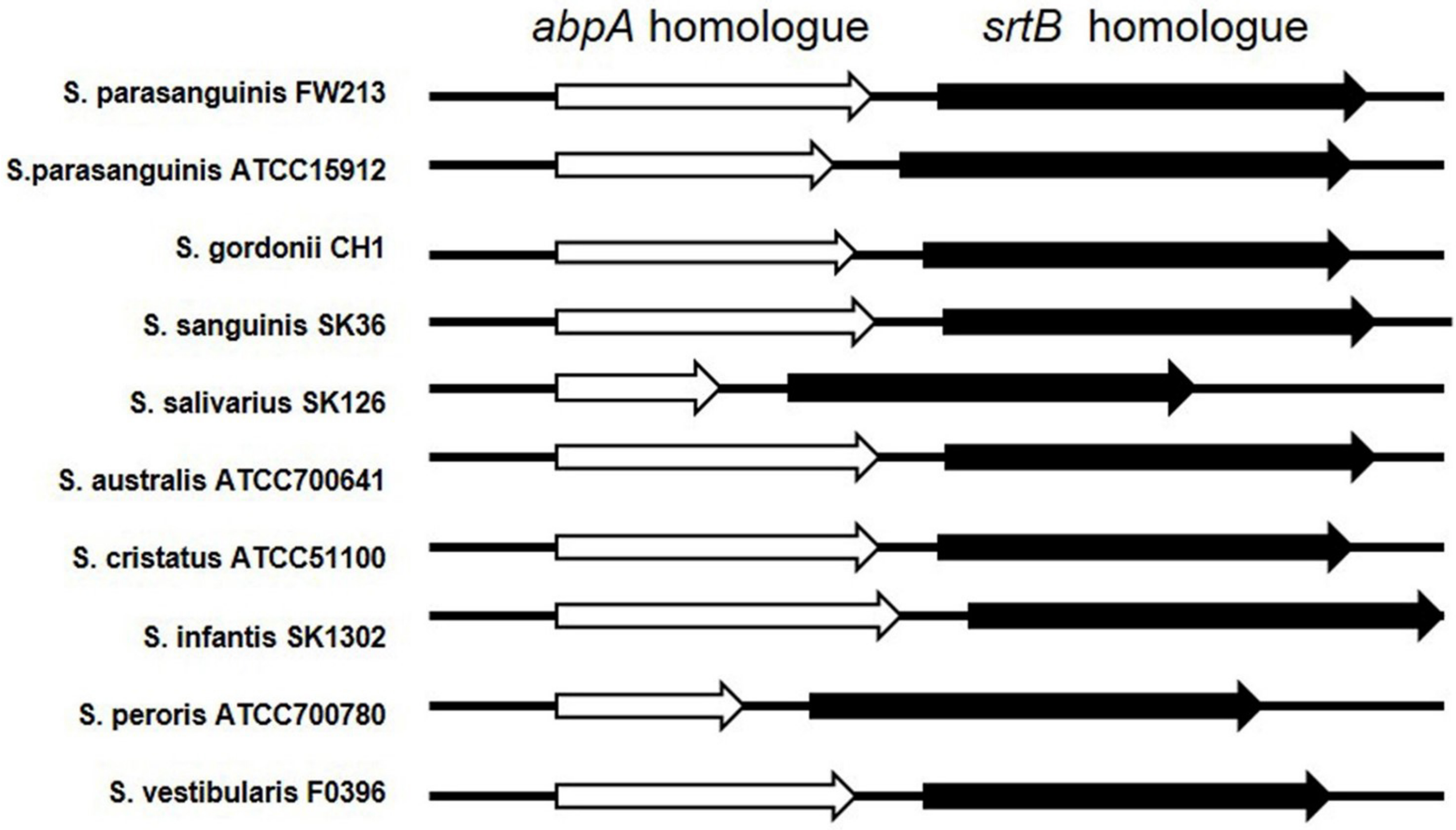

abpA homologue
srtB homologue
S. parasanguinis FW213
S.parasanguinis ATCC15912
S. gordonii CH1
S. sanguinis SK36
S. salivarius SK126
S. australis ATCC700641
S. cristatus ATCC51100
S. infantis SK1302
S. peroris ATCC700780
S. vestibularis F0396

B

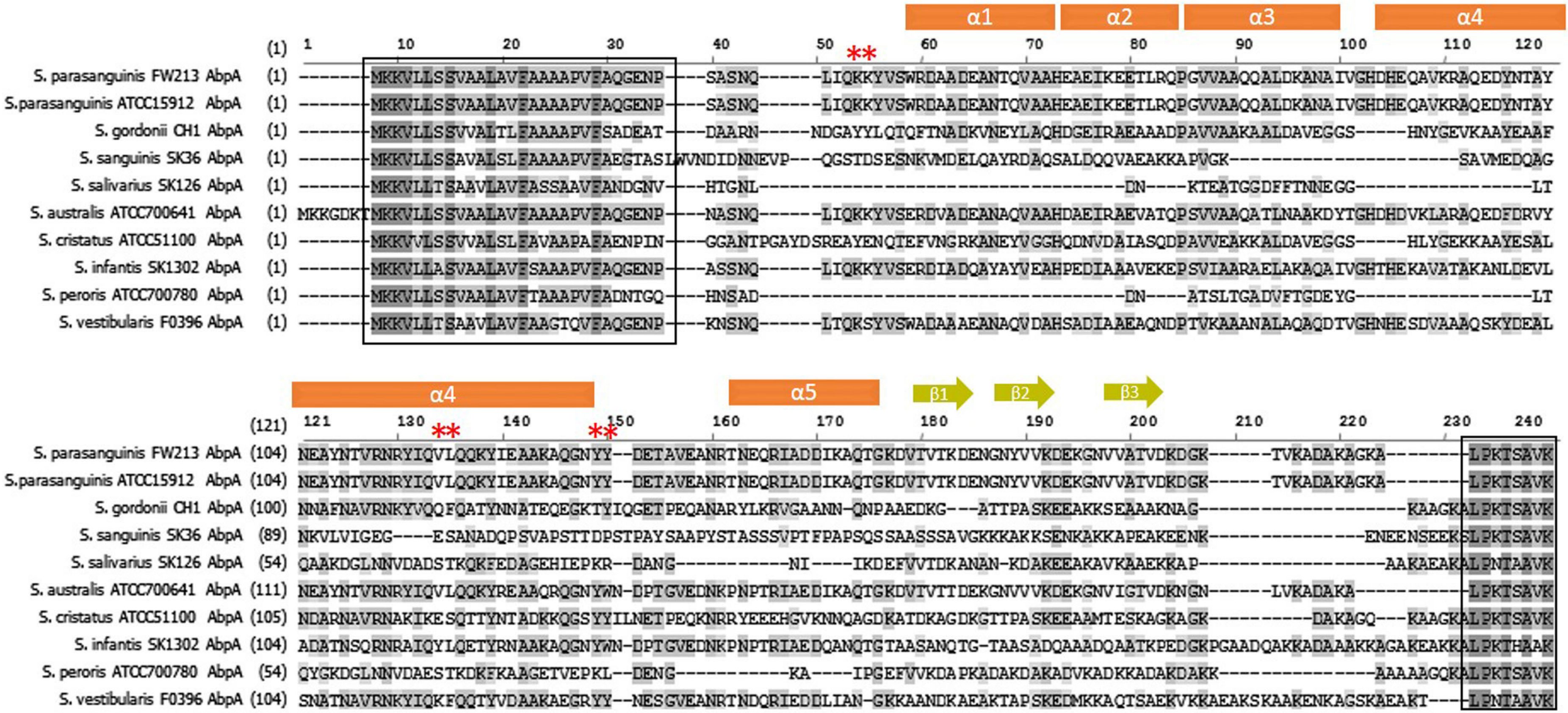

α1
α2
α3
α4
α5
β1
β2
β3
S. parasanguinis FW213 AbpA
S.parasanguinis ATCC15912 AbpA
S. gordonii CH1 AbpA
S. sanguinis SK36 AbpA
S. salivarius SK126 AbpA
S. australis ATCC700641 AbpA
S. cristatus ATCC51100 AbpA
S. infantis SK1302 AbpA
S. peroris ATCC700780 AbpA
S. vestibularis F0396 AbpA

C

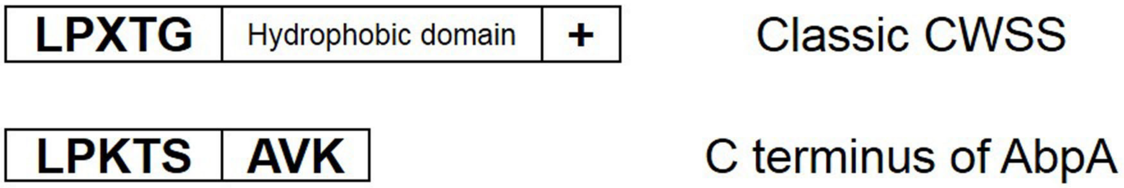

LPXTG
Hydrophobic domain
+
Classic CWSS
LPKTS
AVK
C terminus of AbpA

D

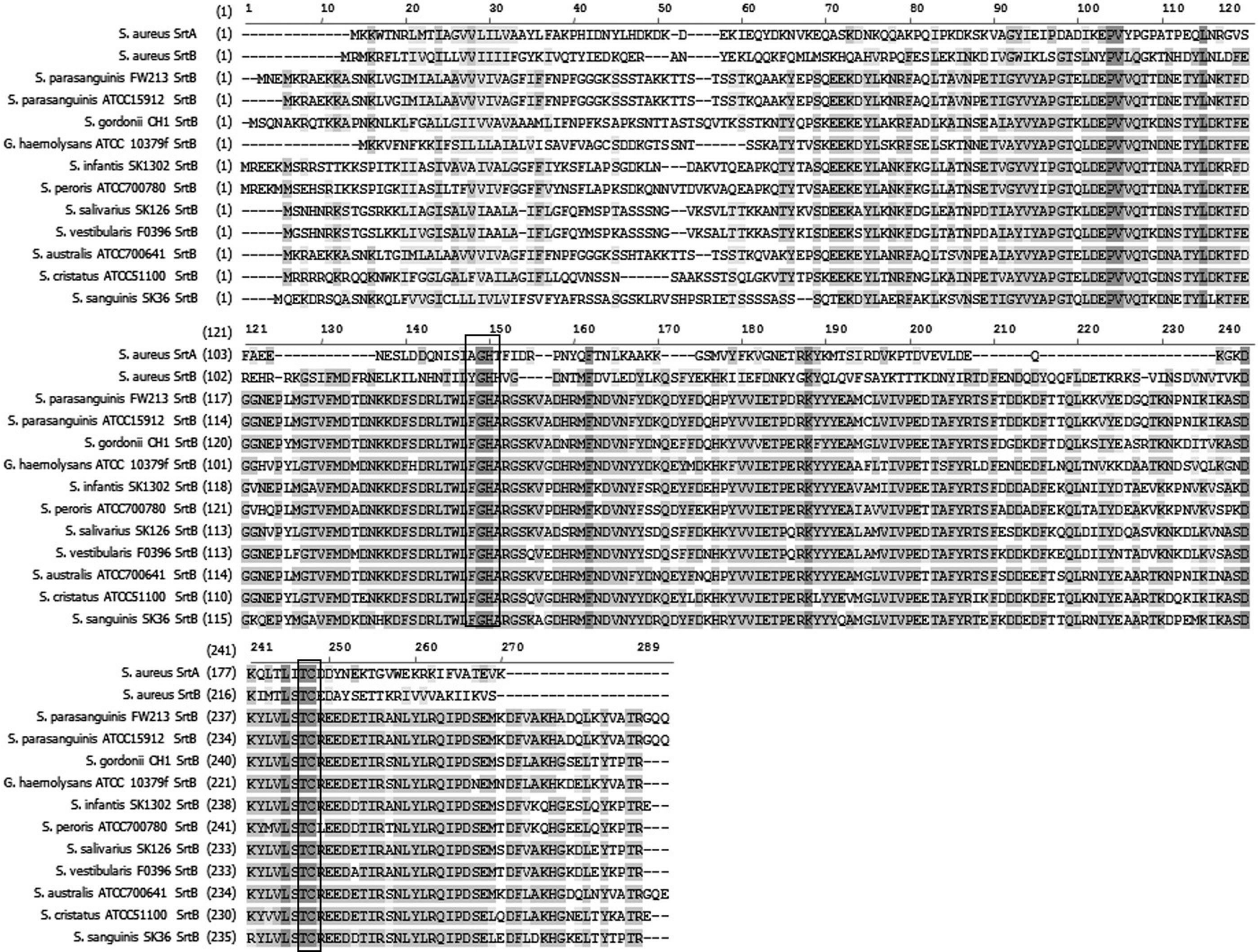

S. aureus SrtA
S. aureus SrtB
S. parasanguinis FW213 SrtB
S. parasanguinis ATCC15912 SrtB
S. gordonii CH1 SrtB
G. haemolysans ATCC 10379f SrtB
S. infantis SK1302 SrtB
S. peroris ATCC700780 SrtB
S. salivarius SK126 SrtB
S. vestibularis F0396 SrtB
S. australis ATCC700641 SrtB
S. cristatus ATCC51100 SrtB
S. sanguinis SK36 SrtB
(1) 1 10 20 30 40 50 60 70 80 90 100 110 120
(121) 121 130 140 150 160 170 180 190 200 210 220 230 240
(241) 241 250 260 270 289

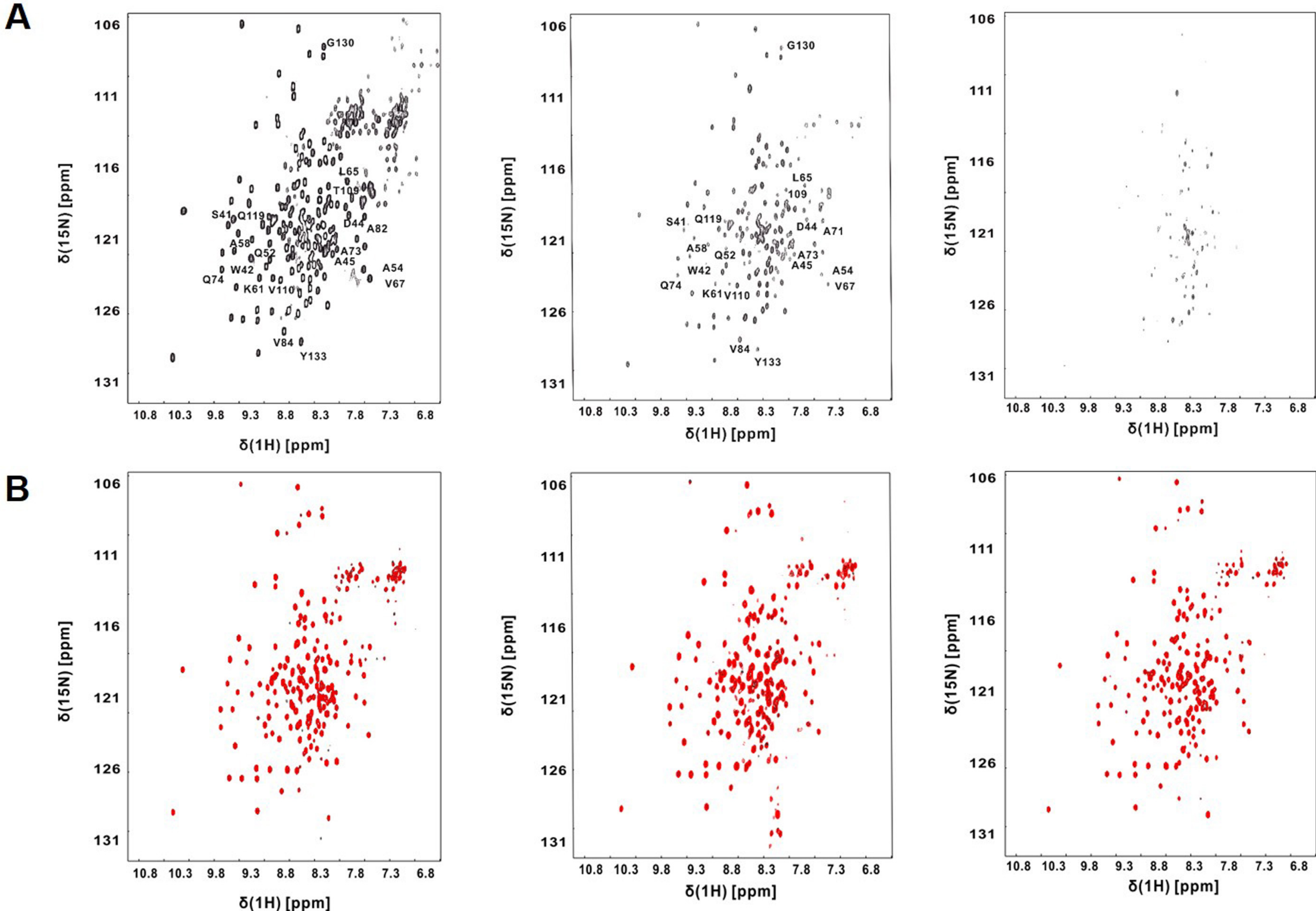
A
B
G130
L65
T109
S41
Q119
D44
A82
A58
Q52
A73
A45
A54
W42
V67
Q74
K61
V110
V84
Y133
G130
L65
109
S41
Q119
D44
A71
A58
Q52
A73
A45
A54
W42
V67
Q74
K61
V110
V84
Y133
δ(15N) [ppm]
δ(1H) [ppm]
106
111
116
121
126
131
10.8
10.3
9.8
9.3
8.8
8.3
7.8
7.3
6.8
